# Tailoring the Variational Implicit Solvent Method for New Challenges: Biomolecular Recognition and Assembly

**DOI:** 10.3389/fmolb.2018.00013

**Published:** 2018-02-12

**Authors:** Clarisse Gravina Ricci, Bo Li, Li-Tien Cheng, Joachim Dzubiella, J. Andrew McCammon

**Affiliations:** ^1^Department of Pharmacology and Department of Chemistry and Biochemistry, National Biomedical Computation Resource, University of California, San Diego, La Jolla, CA, United States; ^2^Department of Mathematics, University of California, San Diego, La Jolla, CA, United States; ^3^Quantitative Biology Graduate Program, University of California, San Diego, La Jolla, CA, United States; ^4^Institut für Physik, Humboldt-Universität zu Berlin, Berlin, Germany; ^5^Soft Matter and Functional Materials, Helmholtz-Zentrum Berlin, Berlin, Germany

**Keywords:** solvation, VISM, implicit solvation, solvation free energy, molecular recognition, binding, solvation free energy of binding, solvent model

## Abstract

Predicting solvation free energies and describing the complex water behavior that plays an important role in essentially all biological processes is a major challenge from the computational standpoint. While an atomistic, explicit description of the solvent can turn out to be too expensive in large biomolecular systems, most implicit solvent methods fail to capture “dewetting” effects and heterogeneous hydration by relying on a pre-established (i.e., *guessed*) solvation interface. Here we focus on the Variational Implicit Solvent Method, an implicit solvent method that adds water “plasticity” back to the picture by formulating the solvation free energy as a functional of all possible solvation interfaces. We survey VISM's applications to the problem of molecular recognition and report some of the most recent efforts to tailor VISM for more challenging scenarios, with the ultimate goal of including thermal fluctuations into the framework. The advances reported herein pave the way to make VISM a uniquely successful approach to characterize complex solvation properties in the recognition and binding of large-scale biomolecular complexes.

## Introduction

The omnipresence of water in all living tissues supports the notion of biochemistry being simply “chemistry in aqueous medium.” As a highly polarizable solvent capable of forming a complex net of hydrogen bonds, water is essential in screening electrostatic forces and in forming specific enthalpic interactions with or between biomolecules (Davis and McCammon, 2000; Ball, 2008). Moreover, water is the inherent counter-player in hydrophobicity, which is perhaps the most important driving force behind self-assembly processes, including biomolecular association and binding (Chandler, 2005; Berne et al., 2009). Indeed, water is crucial for molecular recognition (Levy and Onuchic, 2006; Hummer, 2010; Baron and McCammon, 2013), to such an extent that the free energy of ligand-receptor binding could be dominated not by direct interaction between the ligand and its binding pocket, but by water contributions (Baron et al., 2010; Setny et al., 2010).

Due to intrinsic thermodynamic fluctuations and the many configurations that water molecules can adopt to respond to the perturbation imposed by a solute, hydration (or solvation, if one includes ions into the picture) has a flexible disposition (Ball, 2003). While water can increase its local density in the first solvation shells surrounding hydrophilic solutes, it can also “evaporate” at the vicinity of hydrophobic surfaces, as predicted by theoretical models (Parker et al., 1994; Lum et al., 1999), computer simulations (Huang et al., 2003, 2004, 2005; Choudhury and Pettitt, 2005, 2007), or inferred from experiments (Tyrrell and Attard, 2001; Jensen et al., 2003; Schwendel et al., 2003; Steitz et al., 2003; Poynor et al., 2006). Such dewetting transitions are speculated to speed up the hydrophobic collapse that takes place in folding (ten Wolde and Chandler, 2002; Zhou et al., 2004), self-assembly (Lum et al., 1999; Huang et al., 2003, 2004; Liu et al., 2005) and molecular recognition (Young et al., 2007; Ahmad et al., 2008). In some cases, water might also completely disappear from hydrophobic protein cavities in the unbound state, with important implications for ligand binding (Young et al., 2007; Qvist et al., 2008; Matthews and Liu, 2009; Wang et al., 2011; Krimmer et al., 2017).

The outcomes of water plastic behavior are especially hard to predict when the solute itself also displays a high level of complexity. Thus, while relatively simple models can be used to predict the hydration pattern between two approaching paraffin plates (Lum et al., 1999), such attempt becomes harder for amphiphilic plates (Hua et al., 2009) and practically impossible in face of protein interacting surfaces, whose charge distribution and corrugated topology result in a detailed hydrophobicity/hydrophilicity balance, not to mention their conformational flexibility. Therefore, there is a great interest in developing computational tools to simulate microscopic (water distribution) and thermodynamic properties (solvation free energies) arising from such complexity.

Explicit solvent molecular dynamics (MD) simulations have proved to be a useful approach to describe water plasticity in the context of molecular association and binding, as revealed by simulations capturing capillary evaporation between two hydrophobic surfaces (Huang et al., 2003, 2005; Choudhury and Pettitt, 2005, 2007) and dewetting transitions in the interaction of amphiphilic protein interfaces (Huang et al., 2004; Zhou et al., 2004; Liu et al., 2005). However, explicit solvent MD simulations of large biomolecular systems are expensive and may require an impractical sampling of water configurations in order to obtain converged solvation free energies, unless one relies on the additional use of enhanced sampling techniques and/or perturbation methods.

As an alternative to explicit solvation, there is a great advantage in modeling water as continuum medium, in which microscopic structure and fluctuations are reduced to macroscopically related properties such as dielectric constant, ϵ, and surface tension, γ. Normally referred to as “implicit solvation” or “continuum medium”, methods based on such an approach have significantly lower computational cost and can be used to calculate solvation free energies while avoiding altogether the challenge of getting enough statistical sampling of water configurations.

**Figure 1:**
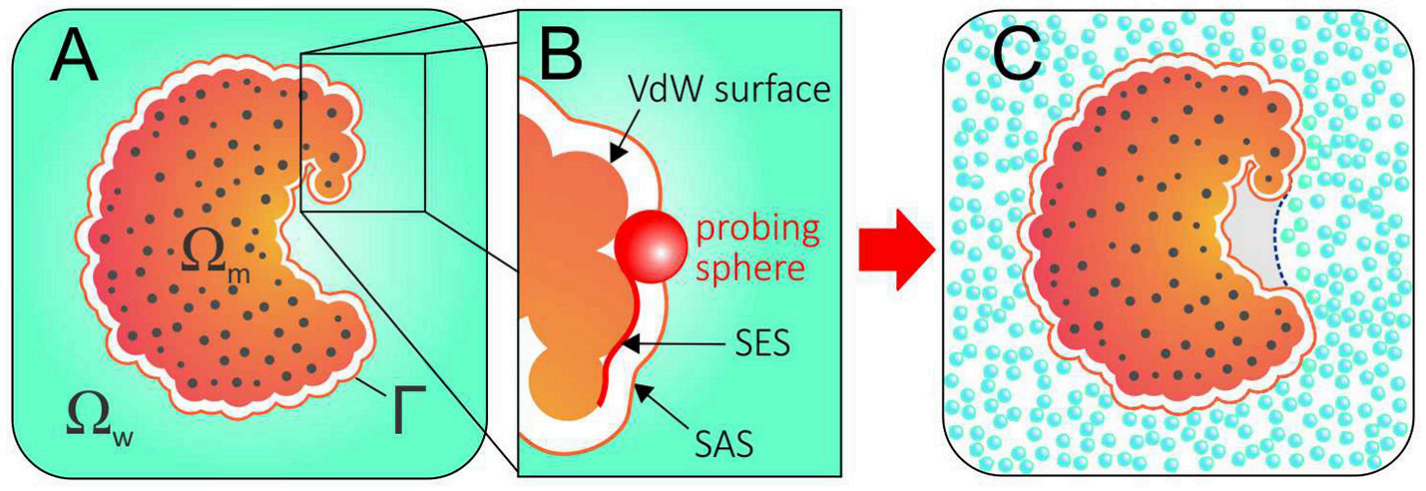
**(A)** The geometry of a system described with an implicit solvent approach. The solute region, solvent region, and solute-solvent interface are denoted by Ω_m_, Ω_w_, and Γ, respectively. **(B)** Solvent Excluded Surface (SES) and Solvent Accessible Surface (SAS) are obtained by rolling a probing ball along the van der Waals surface. **(C)** Conflict between the SAS (solid orange line) and the explicit hydration displayed by a hydrophobic pocket that expels water. A correct implicit solvent description of such pocket would require an alternative solvation boundary (dotted blue line).

Implicit solvation or continuum methods typically split the system into a solute region that is treated explicitly, Ω_m_, and a solvent region that is treated as a continuum, Ω_w_, separated by a dielectric boundary, Γ (Figure 1A). Within this framework, the electrostatic component of the solvation free energy can be modeled by a continuum electrostatic reaction field—obtained by solving the Poisson–Boltzmann equation (Davis and McCammon, 2000; Baker, 2005) or the more simplified Generalized Born model (Bashford and Case, 2000; Feig and Brooks, 2004). Additionally, the hydrophobic contribution arising from first-shell solvation effects can be empirically modeled by surface tension-like coefficients integrated over the solute-solvent surface area (Eisenberg and McLachlan, 1986). Some of the most popular implicit solvent methods estimate the complete solvation free energy by independently calculating and then adding the electrostatic energy to the non-polar solvation free energy, assuming them to be additive (Still et al., 1990; Roux and Simonson, 1999).

In this context, an often neglected question is: what should be the dielectric boundary used in these calculations? While the location and the shape of the solvation interface can significantly impact the results—PB calculations, for instance, are extremely sensitive to the chosen dielectric boundary—implicit solvation methods conventionally employ surfaces that are closely related to the van der Waals surface of the protein (Figure 1B). Therefore, they rely on pre-established solvation interfaces normally *guessed* as solvent accessible surfaces (SAS) and *fixed* during the calculations.

Such an approach is in evident conflict with water's aforementioned plastic behavior and, while successful in many cases, cannot capture dewetting effects or the existence of polymodal hydration. Figure 1C illustrates the discrepant scenario resulting from the use of a SAS as dielectric boundary for a protein whose hydrophobic pocket expels water. More than a didactic example, concave and hydrophobic cavities such as the one in Figure 1C are good representatives of druggable binding pockets. By expelling water, such pockets strongly enhance their affinity for apolar molecules, which do not need to competitively displace water molecules in order to bind (Young et al., 2007; Wang et al., 2011; Krimmer et al., 2017).

This review focuses on the Variational Implicit Solvent Method (VISM), an implicit solvent method that can add water “plasticity” back to the picture by formulating the solvation free energy as a functional of the solvation interface, Γ, and then relaxing the interface toward the solvation free energy minimum (Dzubiella et al., 2006a,b). As such, VISM produces equilibrium solute-solvent interfaces as *output* of the theory while also coupling electrostatic and apolar contributions through the solvation boundary. Noteworthy, our method fits into a longer tradition of functional-based variational methods in condensed matter physics (Lum et al., 1999; Wallqvist et al., 2001; Ramirez and Borgis, 2005; Chen et al., 2010; Zhao et al., 2011; Jeanmairet et al., 2013). We begin by introducing the VISM functional and its combination with a level-set framework (Cheng et al., 2007, 2009b) that allows to deal with the very complex topologies exhibited by proteins and other biomolecules. Next we survey relevant applications of VISM in the context of molecular recognition and binding, highlighting the features that make VISM uniquely able to capture multiple states of solvation and the equilibrium between these states. Finally, we report how VISM can be combined with the MARTINI FF (Marrink et al., 2004, 2007; Monticelli et al., 2008; de Jong et al., 2013) to describe the solvation of coarse-grained proteins, with an example of how this method can be applied to study solvation in encounter complexes. We conclude by outlining the next steps to make VISM an appealing tool not only to model, but also simulate the role of water in large biomolecular assemblies, hopefully in the near future.

## Theory

### The VISM functional

Consider the system displayed in Figure 1A. In VISM, as in other implicit solvation methods, the system is divided in three parts: (i) the solute region, Ω_m_; (ii) the solvent region, Ω_w_; and (iii) a solute-solvent interface, Γ. As usual, the solute region, Ω_m_, contains all the N atoms belonging to the solute molecule, which are located at ***x***_1_, …, ***x***_*N*_ inside Ω_m_ and carry point charges Q_1_, …, Q_N_, while the solvent region, Ω_w_, is treated as a continuum medium. However, instead of using a fixed solvation interface, VISM relaxes an initially guessed interface, Γ, toward the solvation free energy minimum by means of the functional:

(1) $$\begin{array}{l} G\left(\Gamma \right)=\;G_{geometric}\left(\Gamma \right)+G_{dispersion}\left(\Gamma \right)+\;G_{electrostatics}\left(\Gamma \right)\\ G\left(\Gamma \right)=\;\int_{\Gamma }\gamma dS+\rho_{w}\sum_{i=1}^{N}\int_{\Omega_{w}}\text{​}\text{​​}U_{i}\left(\left|x-x_{i}\right|\right)dV+G_{elec}\left(\Gamma \right) \end{array}$$

The first term in Equation (1) is purely geometric and accounts for first-shell solvation effects giving rise to hydrophobicity. In analogy to SASA-methods (Eisenberg and McLachlan, 1986), it consists of an integration of the surface tension, γ, over the solvation interface, Γ. However, the surface tension in VISM's formulation is sensitive to the local shape of the solvation boundary, being defined as:

(2) $$\gamma =\;\gamma_{0}\left(1-2\tau H\right)$$

where γ_0_ is the constant macroscopic surface tension for a planar liquid-vapor interface, *H* is the mean curvature defined as the average of the two principal curvatures, and τ is a curvature correction coefficient (Dzubiella et al., 2006a). Such formulation models the higher surface tension displayed by concave geometries (negative *H*), thus increasing the hydrophobicity of deeply buried pockets as opposed to flat or convex protein surfaces. The curvature correction coefficient, τ, is a fitting parameter that accounts for the relative size of the solvent molecules with respect to the solute local curvature: the larger the size of the solvent molecules, the more sensitive solvent organization will be with respect to the solute curvature, leading to a more pronounced hydrophobic effect.

The second term in Equation (1) accounts for dispersion interactions between water and solute, which are modeled by 12–6 Lennard–Jones potentials, U_i_:

(3) $$U_{i}=4\varepsilon_{i}\left[{\left(\frac{\sigma_{i}}{\left|\;x-x_{i}\right|}\right)}^{12}-{\left(\frac{\sigma_{i}}{\left|x-x_{i}\right|}\right)}^{6}\right]$$

For the *i*th atom of the solute, located at ***x***_*i*_, the interaction potential is integrated over the entire solvent volume, which is represented by a grid with a numeric density pre-factor, ρ_w_. We normally use sub-Å resolution grids, with cells of ~0.3 to 0.5 Å. The solute and solvent Lennard-Jones parameters, ε_*i*_ and σ_*i*_, are borrowed from molecular mechanics force fields (Best et al., 2012; Maier et al., 2015) and the TIP3P water model (Jorgensen et al., 1983).

Finally, the third term in Equation (1), *G*_*elec*_ (Γ), is the electrostatic part of the solvation free energy. This term can be calculated at high level with PB theory (in its linearized or nonlinear forms) (Zhou et al., 2014, 2015), or with the advantageously faster but more approximate Coulomb Field Approximation (CFA) (Wang et al., 2012; Guo et al., 2013):

(4) $$G_{elec}^{CFA}\left(\Gamma \right)\text{​}=\text{​}\frac{1}{32\pi^{2}\epsilon_{0}}\left(\frac{1}{\epsilon_{w}}-\frac{1}{\epsilon_{m}}\right)\;{\int_{\Omega_{w}}\left|\sum_{i=1}^{N}\frac{Q_{i}\left(x-x_{i}\right)}{{\left|x-x_{i}\right|}^{3}}\right|}^{2}\;dv$$

where ϵ_0_ is the vacuum permittivity, and ϵ_*w*_ and ϵ_*m*_ are the relative permittivity for the solvent and the solute, respectively. We highlight the CFA theory because it is common practice to employ CFA for most part of the boundary relaxation, and only switch to PB in the final steps of a VISM calculation (Zhou et al., 2015). Moreover, the studies reviewed in the next section employed CFA whenever electrostatics effects were included in the calculations.

An immediate consequence of the VISM functional is that the apolar (*G*_*geometric*_ + *G*_*dispersion*_) and polar (*G*_*electrostatics*_) components of the total solvation free energy are coupled together through the solvation boundary, Γ. A second consequence is that the VISM functional can be differentiated with respect to the local change of the solvation boundary:

(5) $$\begin{array}{l} \delta_{\Gamma }G\left(\Gamma \right)=2\gamma_{0}\left[H\left(x\right)-\tau K(x)\right]-\;\rho_{w}\sum_{i=1}^{N}U_{i}\left(\left|x-x_{i}\right|\right)\\ \;-\frac{1}{32\pi^{2}\epsilon_{0}}\left(\frac{1}{\epsilon_{w}}-\frac{1}{\epsilon_{m}}\right)\;{\left|\sum_{i=1}^{N}\frac{Q_{i}\left(x-x_{i}\right)}{{\left|x-x_{i}\right|}^{3}}\right|}^{2} \end{array}$$

where *K*(***x***) is the Gaussian curvature, defined as the product of the two principal curvatures, at a point ***x*** on Γ.

**Figure 2:**
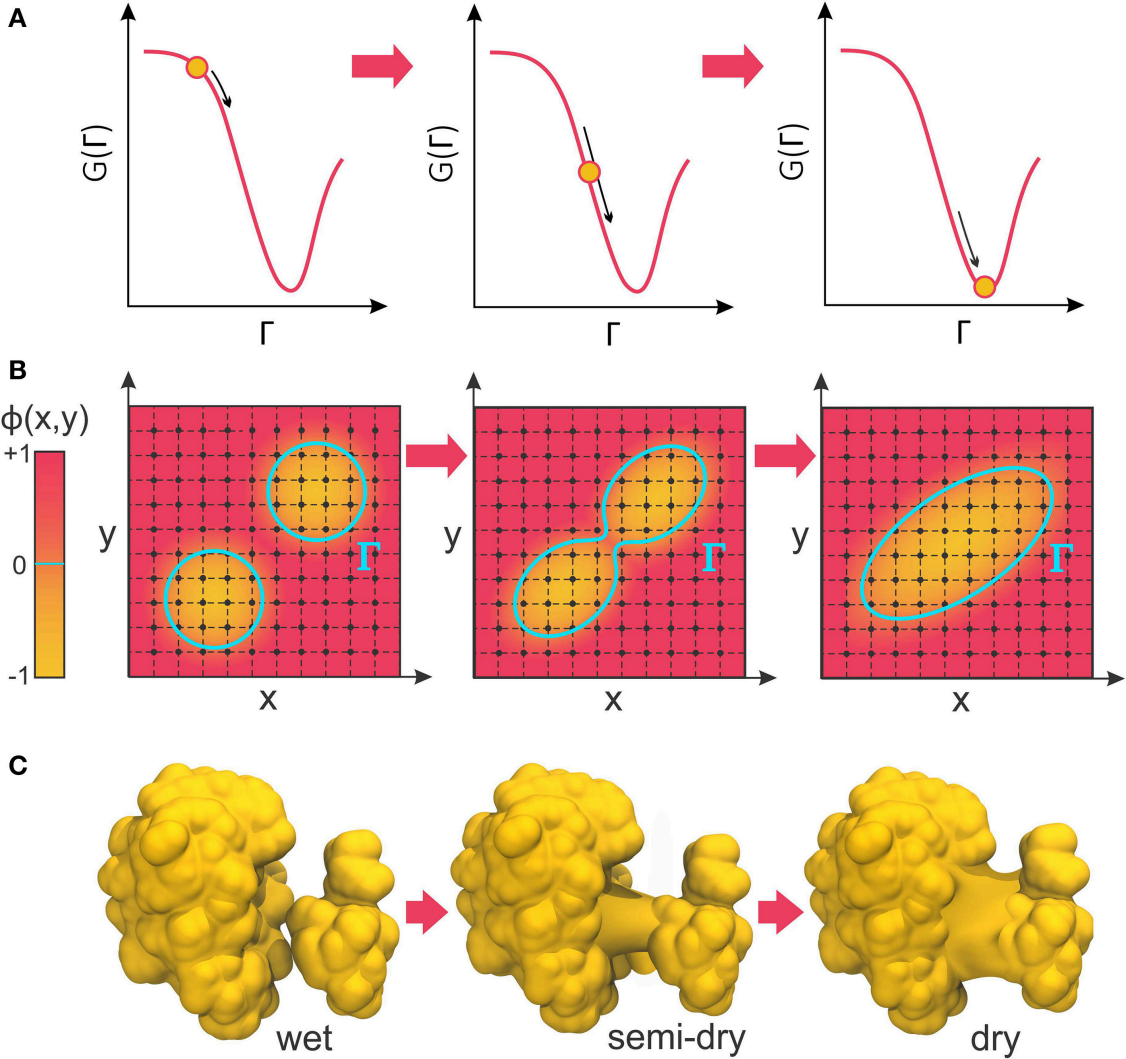
**(A)** VISM calculations minimize the solvation free energy as a functional of the solvation interface, Γ, producing a final solvent-solute boundary that corresponds to a stable solvation state. **(B)** The level-set framework defines the solvation boundary, Γ, as the zero-level set of an auxiliary function, ϕ(x,y). By implicitly manipulating Γ through modification of the level-set function, ϕ(x,y), LS-VISM can easily track topological changes, as when two boundaries merge into one. **(C)** Corresponding example of two solvation interfaces merging as the proteins undergo a dewetting transition.

The negative of this functional derivative, −δ_Γ_*G*(Γ), can be considered an effective “boundary force” whose normal component, pointing from the solute toward the solvent, acts on the boundary interface. In VISM calculations, this force drives the boundary toward a final shape (or solvation state) that minimizes the solvation free energy (Figure 2A). While such minimization can be solved analytically for very simple geometries (Dzubiella et al., 2006a,b), proteins and other biomolecules are far from having topologically simple shapes. Therefore, Cheng et al. have combined VISM to a Level-Set approach, which can numerically find the free-energy minimizing solute-solvent interface for molecules of arbitrary shapes (Cheng et al., 2007, 2009b).

### Level-set VISM (LS-VISM)

In the level-set framework, the solvation boundary is defined as the zero-level set of an auxiliary function ϕ(**x**): Γ = {(**x**)|ϕ(**x**) = 0}. The function ϕ(**x**) – called the level-set function of the surface Γ – is continuous and well-defined at all grid points of the finite computational box encompassing the system (Figure 2B). The solute region, Ω_m_, is defined by points where ϕ(**x**) < 0, while the solvent region, Ω_w_, is defined by points where ϕ(**x**) > 0.

The unit vector **n** at the interface Γ, the mean curvature *H*, and the Gaussian curvature *K* can all be expressed in terms of the level-set function as:

(6) $$n=\;\frac{\nabla \phi \;}{\left|\nabla \phi \right|},\;H=\frac{1}{2}\nabla \;\cdot \;n,\;K\;=\;n\;\cdot \text{ adj}\left(\text{He}\left(\phi \right)\right)n$$

where He(ϕ) is the 3 × 3 Hessian matrix of the function ϕ whose entries are all the second order derivatives $\partial_{ij}^{2}\phi$ of the level set function ϕ, and adj(He(ϕ)) is the adjoint matrix of the Hessian He(ϕ). These properties are necessary for the calculation of the solvation force, −δ_Γ_*G*(Γ) (see Equation 5), which is used in the level-set framework as the normal velocity, υ_*n*_, in solving the level-set equation:

(7) $$\partial_{t}\phi +\;\upsilon_{n}\left|\nabla \phi \right|=0$$

Solution of the level-set equation can be used to determine, in a *steepest descent* manner, the evolution of the level-set function, ϕ(**x**,*t*), and the corresponding motion of the solvation interface, Γ(*t*), defined as the zero-level set of ϕ(**x**,*t*) at each time *t*. Here, “time” corresponds to a minimization step.

The great advantage of the level-set method is that, by implicitly manipulating Γ through the underlying level-set function, ϕ(***x***), it can easily follow shapes that change topology, as for instance when two shapes merge in one (Figure 2B). Therefore, it is an appropriate approach to relax solvation boundaries that merge together as in a desolvated state (Figure 2C) or in the association between two binding proteins. By combining it with the level-set method, Cheng et al. promoted VISM from a toy solvation model to study spherical or cylindrical systems into a competitive solvation method able to deal with systems of more interesting geometries (Cheng et al., 2007, 2009b), as described in the next section.

## LS-VISM applications to molecular association and binding

### Receptor-ligand model systems

**Figure 3:**
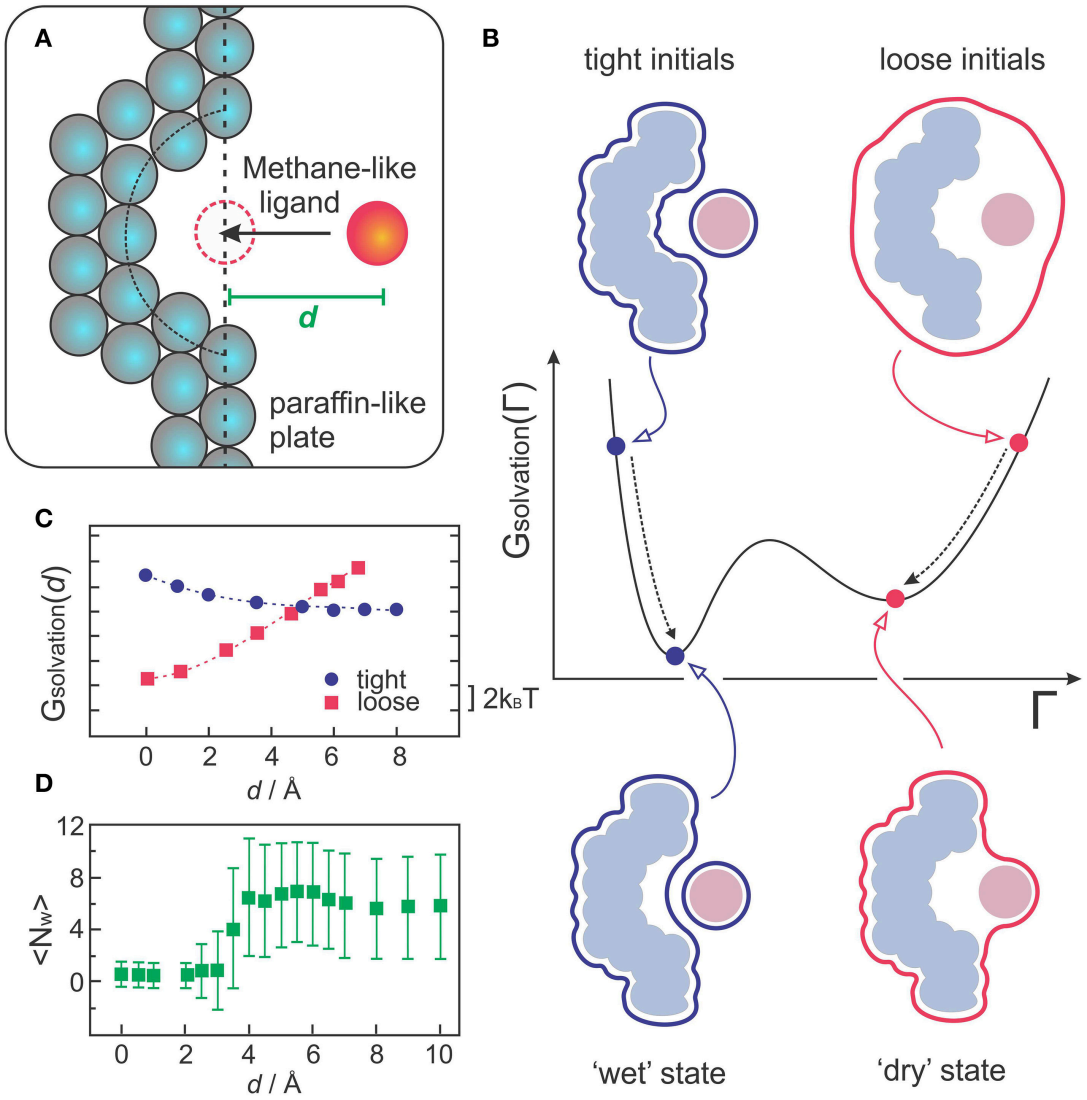
**(A)** Sketch of the generic host-receptor model. The pocket has a hemispherical shape and the methane-like ligand is fixed at a distance *d*, perpendicular to the binding pocket. **(B)** VISM calculations starting from tight or loose initials lead to different minima corresponding to “wet” or “dry” solvation states, respectively. **(C)** VISM Solvation free energy versus the separating distance, *d*, obtained with tight or loose initial surfaces for the host-ligand system depicted in **(A)**. **(D)** Average water occupancy <N_w_> in the pocket from MD simulations. The data in **(C,D)** was originally presented in Cheng et al. (2009a).

VISM was the first implicit solvent method to capture multistate hydration in molecular recognition, as demonstrated with a simple but insightful model of a hydrophobic receptor-ligand system (Cheng et al., 2009a; Setny et al., 2009). The model consists of a paraffin-like plate containing a hemispherical nanoscopic pocket that binds a methane-like ligand (Figure 3A). Despite its apparent simplicity, this system can provide insight into the hypothesis that apolar pockets with concave geometries are particularly prone to capillary evaporation induced by an approaching ligand (Setny, 2007; Young et al., 2007; Ahmad et al., 2008). As such, the solvation properties of this generic host-ligand system have been studied in detail by explicit solvent MD simulations (Setny and Geller, 2006; Setny, 2007, 2008; Cheng et al., 2009a), which captured solvent fluctuations corresponding to more or less hydrated states of the pocket, depending on the proximity of the ligand. While highly hydrated (wet) states predominate at large host-ligand distances, MD simulations revealed that poorly hydrated (dry) states start to become more favorable at a critical distance of ~4 Å, finally predominating over wet states at close distances that do not yet sterically prevent water to occupy the binding interfaces (Cheng et al., 2009a).

Such heterogeneous hydration behavior poses a challenge for VISM, especially considering that the minimization of a free-energy functional with a given initial boundary can only provide one solution describing a static solvent distribution. To deal with this limitation, one can take advantage of the fact that VISM performs only a *local* minimization of the solvation free energy along the “solvation landscape.” Therefore, in systems that allow for heterogeneous hydration patterns, calculations starting from different initial conditions can lead to different local minima, corresponding to distinct equilibrium solvation states (Figure 3B). Cheng et al. performed VISM calculations on this generic host-ligand system starting from several different initial surfaces, some of them tightly wrapping the solutes around their van der Waals surfaces (tight initials), others loosely wrapping the solutes in one single surface (loose initials) (Cheng et al., 2009a). This approach allowed them to capture the bimodal hydration displayed by this hydrophobic cavity and to reproduce the critical ligand distance at where dry states start to predominate over wet states (Figure 3C), followed by a complete dewetting transition, in good agreement with explicit solvent MD simulations (Figure 3D). Moreover, the curvature correction to the surface tension, which is normally absent in SASA-based methods, proved to be crucial to describe dewetting transitions in the concave pocket, with the curvature correction coefficient affecting the onset distance at where polymodal hydration starts (Cheng et al., 2009a; Setny et al., 2009).

### Assembly of the BphC monomer

Wang et al. applied VISM to study the collapse between two domains that form the BphC protein monomer (Wang et al., 2012). Such process can be interpreted as the last step in the folding mechanism of multidomain proteins, with water driving the assembly of individually formed domains into the final globular structure. VISM calculations starting from both tight and loose initials revealed the existence of two “solutions” for the solvation boundary when the domains lie at intermediate separation distances of ~4–14 Å (Wang et al., 2012). These “solutions” correspond to “dry” and “wet” solvation states that indirectly reflect the fluctuating nature of the solvent in equilibrium. With wet states being energetically more favorable than dry ones, a Boltzmann averaging of such discrete states would roughly predict a decreased but not vanishing solvent density in the interdomain region. This prediction is in good agreement with explicit solvent MD simulations of separated BphC domains, showing that water density at separation distances of 6Å becomes 15% lower than in the bulk (Zhou et al., 2004). The fact that this system does not undergo complete dewetting is attributed to favorable electrostatic and van der Waals interactions between BphC and water at the interdomain region. In agreement with MD simulations (Zhou et al., 2004), VISM calculations predicted significant dewetting when the protein charges and dispersion interactions with the solvent were turned off, revealing the importance of coupling polar and non-polar interactions when dealing with biological systems (Wang et al., 2012).

### Binding of p53 to MDM2

**Figure 4:**
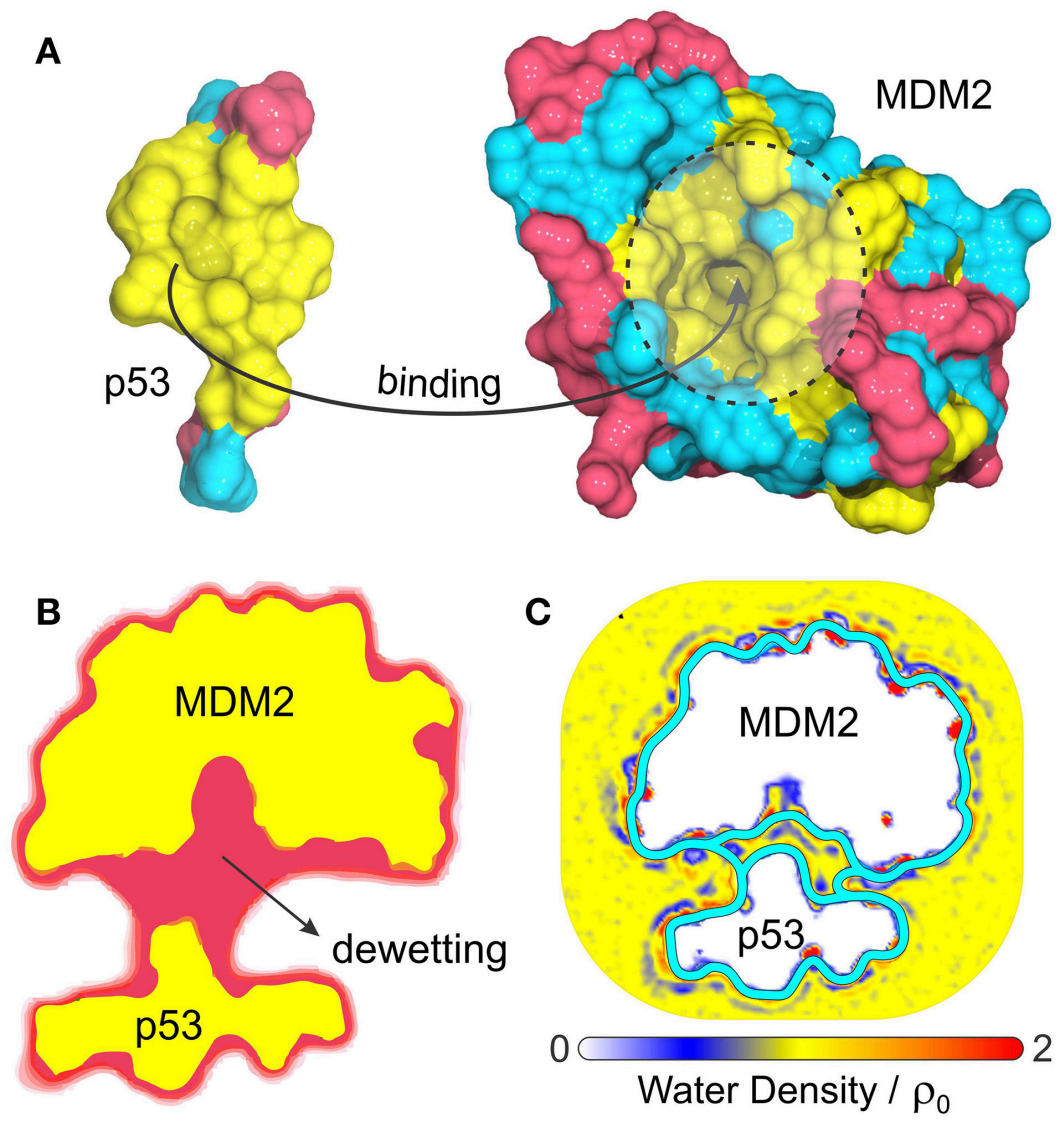
**(A)** Composition and topology of the binding surfaces of the binding domain of p53 and MDM2, with hydrophobic in yellow, charged residues in magenta and neutral hydrophilic residues in cyan. **(B)** Cross-section of the VISM surface (magenta) and molecular surface (yellow) at a separation distance of 14 Å (Guo et al., 2013). **(C)** Water density profile from MD simulations superimposed with the equilibrium VISM surfaces (depicted by thick cyan lines), obtained with loose and tight initials (Guo et al., 2014).

Another interesting biological problem to which VISM has been successfully applied concerns the role of solvation in the binding of tumor-suppressing protein p53 to its repressor, MDM2 (Guo et al., 2013, 2014). p53 plays a vital role in suppressing tumors and disruption of its interaction with MDM2 is the guideline behind many anticancer therapies (Chène, 2003, 2004; Zhao et al., 2013). This system displays a particularly strong hydrophobic character at the p53/MDM2 binding interface (Kussie et al., 1996), with MDM2's binding pocket comprising a concave apolar patch with few hydrophilic residues lying at the edges (Figure 4A). As such, p53-MDM2 complex provides an excellent example of a biologically relevant system whose molecular association is likely to involve dewetting transitions or at least heterogeneous hydration.

Guo et al. have used VISM to estimate the solvation behavior arising from the delicate interplay between complicated geometry, hydrophobicity and polar interactions at the binding interfaces of p53 and MDM2 (Guo et al., 2013, 2014). As a result, VISM calculations of the two proteins separated by distances as large as 14 Å revealed significant water depletion inside the binding pocket and in the inter-domain region (Figure 4B) (Guo et al., 2013). This was found to be consistent with explicit solvent MD simulations of the two proteins separated by 12 Å, which revealed capillary evaporation at the bottom of MDM2's binding pocket, with the first solvation layers forming near its entrance (Figure 4C) (Guo et al., 2014). Additionally, the solvation free energy of binding estimated by VISM (237.3 kcal/mol) was in relatively good quantitative agreement with explicit water FEP calculations (306.7 kcal/mol), especially considering the large size (and associated errors) of this system (Guo et al., 2013). To conclude, dewetting transitions captured by VISM calculations are not only realistic, but they might also contribute for fast kinetics of binding between p53 and MDM2. Thus, by efficiently describing solvent behavior of this type of system, VISM could contribute to develop solvation-driven strategies of controlling the association of protein complexes with relevant impact in biology and health.

### ^M^VISM and solvation for coarse-grained complexes

Combining VISM with a coarse-grained model for the solute is an interesting step to push VISM toward large-scale applications and eventually merge it with molecular dynamics simulations. Recently, we adapted VISM to produce solvation free energies for “martinized” proteins (Ricci et al., 2017). The MARTINI model is a well-established meso-scale force field for modeling large molecular systems, which replaces groups of atoms by interaction centers commonly referred to as “beads,” based on an approximate 4-to-1 mapping (Marrink et al., 2004, 2007; Monticelli et al., 2008).

For this purpose, the main adaptation of the VISM functional consisted in replacing atomistic Lennard-Jones parameters, ε_*i*_ and σ_*i*_, by the coarse-grained LJ parameters reported in Martini 2.1 (Marrink et al., 2007; Monticelli et al., 2008). Application of this new method, denominated ^M^VISM, to estimate the solvation free energies of six different proteins revealed a good qualitative agreement between the fully coarse-grained approach and the atomistic version obtained with the original LS-VISM. We also found the solvation free energies obtained with ^M^VISM to be significantly underestimated due to un-optimized (too favorable) Lennard-Jones interaction energies between solute and water (Ricci et al., 2017). Overestimation of van der Waals interactions is characteristic of MARTINI, as reported in hydration of organic compounds (Marrink et al., 2007), protein-protein binding (Stark et al., 2013) and aggregation of polysaccharides (Schmalhorst et al., 2017). This trait stems from the fact that the force field was originally developed to model highly apolar environments such as lipid bilayers. In fact, increasing the strength of LJ interactions is a way of compensating for (i) the simplistic treatment given to electrostatic interactions in the Martini model, and (ii) the reduced number of degrees of freedom in Martini water, which can significantly reduce entropically-driven hydrophobic attraction between apolar molecules. In ^M^VISM, overestimation of van der Waals interactions could be easily fixed by a simple downscaling of the Martini ε_*i*_ values, which brought solvation energies from the fully coarse-grained approach to fairly good quantitative agreement with the ones from the atomistic approach, while also providing a better partition of the solvation free energy among the coupled energy terms (hydrophobic, Lennard-Jones, and electrostatics) (Ricci et al., 2017).

**Figure 5:**
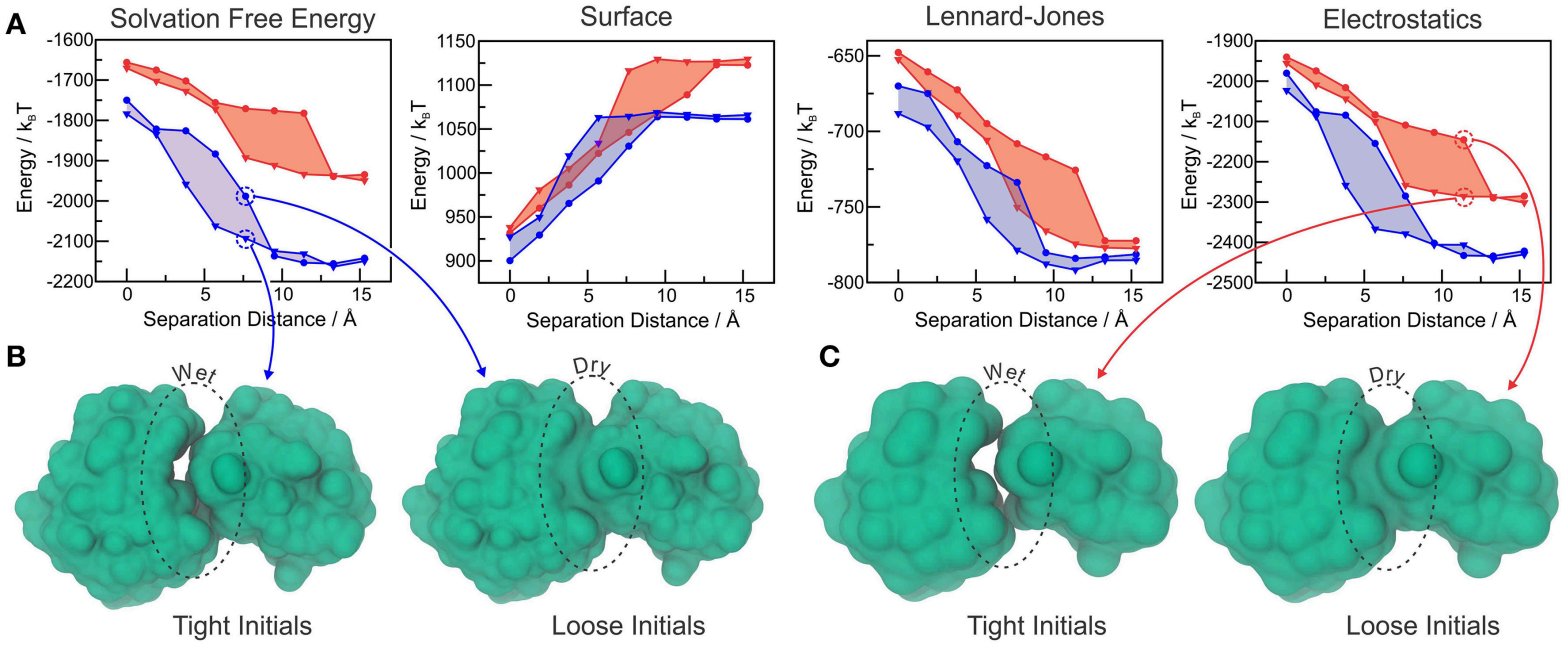
**(A)** Total solvation free energy, surface, Lennard-Jones and electrostatic solvation energies for Barstar-Barnase at different separation distances, calculated with LS-VISM (blue) or ^M^VISM (red), starting from tight (triangles) or loose (circles) initial surfaces. **(B)** Solvation surfaces obtained with LS-VISM, at *d* = 7.6Å. **(C)** Solvation surfaces obtained with ^M^VISM, at *d* = 11.4Å. Adapted from Ricci et al. (2017).

We further tested ^M^VISM's ability to describe hydration in the tight-binding barnase-barstar complex, which has achieved its extremely fast kinetics of binding by means of optimized electrostatic interactions (Lee and Tidor, 2001). Figure 5A shows the solvation free energies obtained with coarse-grained (^M^VISM) or atomistic (LS-VISM) calculations of the barstar-barnase complex with separation distances ranging from 0 (native complex) to 15 Å. As compared to atomistic solvation energies, the coarse-grained energies are not as favorable, mainly due to the electrostatic term. Overestimation of the electrostatic solvation energy is very likely a consequence of (i) polar beads carrying no partial charges in Martini2.1, and (ii) the point charges carried by charged beads being buried deeper in the solute interface due to the large size of the beads.

The hysteresis in the energies obtained with loose or tight initial surfaces evidence the existence of multiple solvation states, some of which are illustrated in Figures 5B,C. As expected, tight initial conditions produce states that are systematically more hydrated as compared to the states obtained with loose initials, with larger discrepancies occurring for intermediate separation distances. Interestingly, both methods predict the wet encounter pathway to be significantly more stable than the dry one, as a result of favorable electrostatic interactions of the binding interfaces with water. This finding indicates that, despite the simplifications imposed by the Martini model in the treatment of electrostatic interactions, ^M^VISM can successfully predict the highly hydrated nature of electrostatically-optimized association between barstar and barnase.

### Final considerations

In this review, we focus on the level-set VISM (LS-VISM) and its ability to capture the plastic behavior displayed by water in molecular binding and protein-protein association. Such ability distinguishes VISM from more traditional implicit solvent methods that rely on a fixed and pre-established solvation boundary, which are intrinsically incapable of capturing dewetting events.

We surveyed a few relevant applications of LS-VISM in the study of receptor-ligand hydrophobic binding, hydrophobic assembly of BphC protein, and dewetting effects that govern the association between p53 and MDM2. While these studies display good agreement with explicit solvent MD simulations, it is worth noting that they were performed with a relatively simple treatment of electrostatic interactions (CFA) or no electrostatic treatment at all in the case of hydrophobic ligand-host studies. More accurate results could be obtained by employing a Poisson-Boltzmann treatment of electrostatic interactions, as implemented in the current version of LS-VISM, also available in a public software package (Zhou et al., 2015).

In the last example, we showed how LS-VISM can work in combination with Martini2.1 to predict the hydration behavior in the association of a relevant biological complex (barnase-barstar). While encouraged by this success, we are aware that ^M^VISM could be further improved by using newer and more sophisticated versions of Martini, which provide better electrostatic description and attempt to deal with the problem of superestimated LJ interactions and over aggregation. We are currently testing the more recent “polarizable” Martini2.2P (de Jong et al., 2013) and looking forward to test a new Martini that should be released in the near future (personal communication).

The use of a coarse-grained model for the solute is a sensible strategy to combine VISM with MD simulations, which typically require many thousands of integration steps if one desires to sample rare events such as diffusion-controlled molecular binding while still keeping track of conformational dynamics and solvation plasticity. Unfortunately, relaxation of the solvation boundary with LS-VISM is still too slow (minutes to hours) and poses the last remaining obstacle preventing VISM from being efficiently combined with MD simulations. To deal with the problem of speed, we are currently working on a new boundary-relaxing algorithm capable of approximating the boundary solutions produced by the level-set framework with significantly lower computational cost. We expect this approach to facilitate the development of a hybrid MD-VISM method to not only model, statically, but actually *simulate* solvation in the assembly of large biomolecular complexes using the VISM framework.

## Author contributions

All authors contributed to the design, organization, and review of the manuscript. CR took the lead in writing the manuscript and prepared the figures.

### Conflict of interest statement

The authors declare that the research was conducted in the absence of any commercial or financial relationships that could be construed as a potential conflict of interest.
